# Editorial: Overcoming challenges in microbial immunology: 2022

**DOI:** 10.3389/fimmu.2024.1436631

**Published:** 2024-06-17

**Authors:** Beatrice Ondondo

**Affiliations:** Immunology Department, University Hospitals of Leicester National Health Service (NHS) Trust, Leicester, United Kingdom

**Keywords:** short chain fatty acids (SCFAs), traditional Chinese medicine, rheumatoid arthritis, cardiovascular disease (CVD), Q-fever, sepsis, cutaneous T cell lymphoma (CTCL), phagolysosome pathway

As a rapidly expanding field, Microbial immunology faces unprecedented challenges: discovery of new microbes; treatment of drug-resistant microbes; and determining how microbes shape the immune landscape during infection, cancer, and autoimmunity. Furthermore, microbes may indirectly influence the development, progression, and prognosis of chronic non-communicable diseases. This Research Topic highlights the complex interactions of microbes with the immune system and shows that specific targets of such interactions hold promise for novel therapeutic and vaccination strategies. Collectively they point to areas for further development in the field and provide a focus for future research.

Strong links exist between the microbiome and cancer (1–3), and imbalances in the gut microbiome are associated with various chronic diseases including obesity, airway inflammation, colitis, some digestive disorders and cardiovascular disease (CVD) (4, 5). Gut microbiota produce bioactive metabolites including trimethylamine, trimethylamine N-oxide, short-chain fatty acids (SCFAs), and bile acids, which may have a link to the aetiology of CVD (6). Luqman et al. provide an overview of the intricate links between gut microbiota, their metabolites, and the development of CVD. They focus on how intestinal dysbiosis promotes CVD risk factors such as heart failure, hypertension and atherosclerosis, and potential therapeutic interventions using gut microbes and their metabolites. SCFAs regulate the immune system and modulate inflammatory responses (7, 8) through their action on various cell types and can impact the prevention and treatment of disease. Liu et al. summarise the different mechanisms through which SCFAs act in cells with particular emphasis on their regulatory role in innate and adaptive immune systems. They highlight the role of SCFAs in regulating allergic airway inflammation, colitis, and osteoporosis through influencing the immune system, and suggest that metabolic regulation can inform treatment options.

The established relationship between gut microbiota and rheumatoid arthritis (RA) (9) suggests that therapeutic approaches for RA may include the active modulation of gut microbiota. Traditional Chinese medicine (TCM) has been suggested to regulate immunity, reduce inflammation and improve quality of life (10) by exerting its effects on the gut microbiota. Liang et al. explore the complex relationship between TCM and gut microbiota not only in the context of treating RA, but also the role of gut microbiota in its pathogenesis and prognosis. They further elucidate mechanisms to utilize TCM in the treatment and prevention of RA by regulating gut microbiota and provide an evidence-based rationale for investigating microbiota-targeted intervention by TCM. In recognition of the link between oral health and general well-being, Xu et al. provide a detailed review on how interactions of oral microbiota with the host can lead to alveolar bone resorption. They highlight various mechanisms through which *P. gingivalis*- and *F. nucleatum*, besides causing periodontitis, disrupt the host osteoimmune mechanisms leading to alveolar bone resorption and describe the immunophenotypes observed in host periodontal tissues during pathological conditions.

In contrast to the extensive prior focus on the relationship between gut microbiota and cancer, Wu et al. explore the role of intra-tumoral microbiota in cancer onset, progression, and therapy. They provide insight on how microbiota within the tumour microenvironment exert immunomodulatory effects to promote inflammation and directly compromise anti-tumour immunity. Their review highlights the potential for novel cancer therapies targeted to specific intra-tumoral microbiota and calls for further research to advance this promising field. Using the murine EL4 model, Dey et al. show that phototherapy in conjunction with antibiotic treatment can modulate skin microbiota and alter the course of cutaneous T-cell lymphoma. They demonstrate that the extent of microbial colonisation of the skin correlates with disease severity and tumour growth, and that antibiotics can significantly delay tumour occurrence, leading to increased survival. They found that antibiotics enriched the skin microbiome with commensal Clostridium species while significantly reducing facultative pathogens and *Staphylococcus aureus*. Reduction of pathogenic microbes may curtail the chronic inflammation caused by skin-homing T cells: a prominent characteristic of cutaneous T-cell lymphoma. Their findings may support the development of novel therapeutic agents to modulate the microbial milieu in patients with cutaneous T-cell lymphoma.

Taya et al. highlight the importance of developing new classes of antimicrobial agents that can complement host-directed therapies (HDT) to overcome the significant problem of emerging drug-resistant microbes. If successful, HDT may be extremely useful during overwhelming sepsis before identifying the causative microbes. Taya et al. provide insights on how the phagolysosome pathway, a first line of defence in the innate immune system, can be modulated for HDT despite the myriad of strategies employed by microbes to escape and survive this pathway (11, 12). The abundance of detailed molecular biological analyses of the phagolysosome system (13–15) provide key information for the development of drugs that target various points of action in this pathway including phagocytosis, phagosome maturation, fusion with lysosomes and lysosome acidification. Unlike the stratification of sepsis patients based on genomic and transcriptome data (16, 17), studies utilising immune profiles at protein expression level are scarce. Tang et al. report on the immune landscape of sepsis and a prediction model that classifies patients into three distinct immune endotypes, which are characterised by different survival rates. By comparing signatures of innate and adaptive immune function in sepsis patients to healthy controls, they discover a dysregulation-type immune endotype associated with a lower survival rate owing to significant impairment of innate and adaptive immunity and increased inflammation. Their study suggests that septic immune endotypes could inform future development of personalized therapies.

The concept of trained immunity has been reported in natural infection and following vaccination where it may enhance immunity against microbes or cause aberrant inflammation in certain situations (18). Using flow cytometry to profile *ex vivo* recall responses, Raju Paul et al. demonstrate the occurrence of trained innate immunity following natural exposure to *Coxiella burnetii*, the causative agent of Q fever. Their study reveals long-term persistence of CD14+ monocytes producing elevated levels of IL-6, IL-1β and IL-8 in individuals pre-exposed to *C. burnetii*. If these cells exert sustained protection against Q fever, or significantly alter the course of disease, they may hold useful clues for vaccines against Q fever.

In conclusion, this Research Topic elucidates how microbiota (intestinal, oral, skin, and intra-tumoral) influence disease progression through modulation of innate and adaptive immunity. It provides a glimpse at possible innovative approaches to harness microbe-host-interactions for the treatment of cancer, infections, and chronic diseases. Research that optimises non-conventional therapies such as faecal transplantation, TCM, HDT, and dietary treatments will propel this field forward.

## Author contributions

BO: Conceptualization, Writing – original draft, Writing – review & editing.
